# Re-Emergence of Dengue Virus Type 3 in Canton, China, 2009–2010, Associated with Multiple Introductions through Different Geographical Routes

**DOI:** 10.1371/journal.pone.0055353

**Published:** 2013-02-06

**Authors:** Huiying Liang, Lei Luo, Zhicong Yang, Biao Di, Zhijun Bai, Peng He, Qinlong Jing, Xueli Zheng

**Affiliations:** 1 Department of Primary Public Health, Canton Center for Disease Control and Prevention, Canton, Guangdong Province, People’s Republic of China; 2 Department of Communicable Disease Control and Prevention, Canton Center for Disease Control and Prevention, Canton, Guangdong Province, People’s Republic of China; 3 School of Public Health and Tropical Medicine, Southern Medical University, Canton, Guangdong Province, People’s Republic of China; Centers for Disease Control and Prevention, United States of America

## Abstract

**Background:**

Endemic dengue virus type 3 (DENV-3) infections have not been reported in Canton, China, since 1980. In March 2009, DENV-3 was isolated for the second time, occurring about 30 years after the previous circulation. In August, 3 other cases emerged. One much larger outbreak occurred again in 2010. To address the origin and particularly to determine whether the outbreaks were caused by the same viral genotype, we investigated the epidemiological and molecular characteristics of the introduction, spread and genetic microevolution of DENV-3 involved.

**Methodology/Principal Findings:**

Three imported cases (index-1,2,3) separately traveled back from Vietnam, India and Tanzania, resulted in 1, 3 and 60 secondary autochthonous cases, respectively. In autochthonous cases, 64.6% positive in IgM anti-DENV and 18.6% in IgG from a total of 48 submitted serum samples, accompanied by 7 DENV-3 isolates. With 99.8%, 99.7%, and 100% envelope gene nucleotidic identity, 09/GZ/1081 from index-1 and endemic strain (09/GZ/1483) belonged to genotype V; 09/GZ/10616 from index-2 and endemic strains (09/GZ/11144 and 09/GZ/11194) belonged to genotype III Clade-A; and 10/GZ/4898 from index-3 and all four 2010 endemic DENV-3 strains belonged to genotype III Clade-B, respectively.

**Conclusions/Significance:**

Both epidemiological and phylogenetic analyses showed that the 2010 outbreak of dengue was not a reemergence of the 2009 strain. Introductions of different genotypes following more than one route were important contributory factors for the 2009–2010 dengue epidemics/outbreaks in Canton. These findings underscore the importance of early detection and case management of imported case in preventing large-scale dengue epidemics among indigenous peoples of Canton.

## Introduction

Dengue virus, a member of the genus *Flavivirus,* family *Flaviviridae,* has four serologically related but genetically distinct virus serotypes (DENV-1 to DENV-4) [1]. Infection with one of the four serotypes can result in a range of clinical manifestations from asymptomatic infection to dengue fever (DF) and the severe disease dengue hemorrhagic fever (DHF)/dengue shock syndrome (DSS) [2]. The geographic expansion of dengue and its increased incidence is increasingly recognized as a public health challenge that is currently unmet by licensed vaccines, specific therapeutic agents, or efficient vector-control strategies [3]. Globally, 2.5 to 3 billion people living mainly in tropical and subtropical regions are estimated to be at risk of infection with dengue viruses. It is estimated that there are at least 100 million dengue infections annually and half a million DHF cases which require hospitalization. Of the latter, the mortality rates average 5%, with 25,000 deaths each year, mostly among children [4].

In China, historical epidemics of dengue-like illness have been documented before 1950 [5]. However, etiological and epidemiological investigations were not carried out on these epidemics [6]. From 1950 to 1978, outbreaks of dengue-like illness were not reported in China. A sudden outbreak of dengue in Foshan city (contiguous to Canton) in 1978, followed by expansion into Canton, started the re-emergence of dengue in China [5], [6]. Since then, outbreaks and epidemics of dengue, with cases of DHF, have been reported sequentially in Canton [7], [8]. Accounting for nearly 56% of the dengue cases in mainland China, Canton has now been a representative city suffering annual DENV transmission [7]. However, up to now, dengue in Canton is still characterized as an imported disease, without recognized evidence supporting the presence of its epidemic foci. Like other China cities, such as Dongguan [9], Shantou [10], Ningbo [11], and Yiwu [12], imported dengue cases are also regarded as playing a key role in initiating outbreaks or epidemics in Canton [13].

All four serotypes have been sequentially circulated in Canton from 1978 to 2008. Beginning in 2000, DENV-1 has been implicated as the causative agent in dengue outbreaks and epidemics. Although DENV-3 was detected sporadically from imported cases, no DENV-3 related epidemic occurred until the first endemic DENV-3 was isolated in March 2009 since 1980. At the start of August, DENV-3 isolates were again reported. Then in 2010, one much larger dengue outbreak occurred in Canton again, in which DENV-3 virus was identified as the major serotype, too. However, it is still unclear which geographical route allowed the virus entry in Canton and how it has genetically changed since then. Again, the concern as to whether the dengue outbreaks had become endemic in Canton arose.

To address the origin and particularly to determine whether the outbreaks were caused by the same viral genotype, we investigated the epidemiological and molecular characteristics of the introduction, spread and genetic microevolution of DENV-3 involved.

## Materials and Methods

### Ethics Statement

Serum specimens of patients were sampled for dengue diagnostic and surveillance purposes (under medical prescription by a physician) with ethical approval from the Ethics Committee of the Canton Center for Disease Control and Prevention (Canton CDC). The use of biological samples and the collection of information were performed in accordance with the China regulations. Written informed consent was obtained from all patients. However, the identity of all participants was kept confidential.

### Patients and Samples

All patients were identified from passive surveillance when seeking medical services or recognized by active searches that were reported to the Notifiable Infectious Disease Report System (NIDRS) within 24 hours after clinical diagnosis. Especially, confirmation of dengue virus infection will prompt an immediate reaction of public health authorities to reduce the risk of further spread of the virus, including active case finding in the neighbour-hood of the case’s residence and in other areas visited by the case. A standardized questionnaire was adopted by local epidemiologist to collect the demographic, clinical, and epidemiological information from each case through a face-to-face interview.

Blood samples of suspected cases were taken synchronously on this occasion and transported (at 4°C) to the Canton CDC laboratory for serologic diagnosis and etiologic agent identification. Human serum samples used in this study were derived from confirmed dengue cases submitted to Canton CDC during 2009 and 2010. Case definition, serological tests, and viral isolation and identification from sera samples conformed to the unified Diagnostic Criteria for Dengue (WS216-2008) enacted by the Chinese Ministry of Health. Autochthonous and imported dengue cases were defined depending on the travel history to dengue-endemic or -epidemic regions within 3–14 days before the onset of the illness.

### IgM- and IgG-capture Enzyme-linked Immunosorbent Assay (ELISA)

A non-serotype-specific Dengue Duo IgM- and IgG-Capture ELISA Kit (PanBio, Windsor, Australia) was used to serologically diagnose infections according to the manufacturer’s instructions [13].

### Virus Isolation and RNA Extraction

*Ae.albopictus* C6/36 cells, passaged in Dulbecco’s modified Eagle’s medium (DMEM) supplemented with 10% fetal calf serum (FCS), were used for virus isolation. The cell monolayer was incubated with 200 µL of serum sample at 30°C for one hour in a 5% CO_2_ incubator before 2 mL of DMEM supplemented with 2% FCS was added. The cultures were incubated for seven days and observed daily for cytopathic effects (CPEs). At the end of the seven-day incubation, the supernatant was harvested and clarified by centrifugation at 2,500 rpm before incubation onto a fresh cell monolayer. The C6/36 *Ae.albopictus* cell supernatants were then preserved at −80°C [14]. Viral RNAs were extracted from the supernatants of cell culture, to which serum from dengue patients had been inoculated, using a QIAamp Viral RNA mini kit (Qiagen, Hilden, Germany) according to the manufacturer’s protocol [15].

### Reverse transcription–polymerase Chain Reaction (RT-PCR)

Amplification of the *E* gene was then performed using the OneStep RT-PCR® Kit (TaKaRa, Shiga, Japan) and three designed oligonucleotides primer pairs [D3E/622F (5′-AGA CAT TGA CTG GTG GTG C-3′) and D3E/1355R (5′-TGA TGA CGG TGT ATT TGA GG -3′); D3E/1243F (5′-CGG TTG TGG TTT GTT TGG-3′) and D3E/1876R (5′-CTG CGT TTC TGA GAC TTC TTT C-3′); D3E/1711F (5′- TAC CGC ACT GAC AGG AGC-3′) and D3E/2684R (5′- CCA CAA CTA CCG TTA ATT TGA T-3′)] in order to produce three overlapping fragments covering the complete E gene. Genomic RNA of the isolates was reverse transcribed into cDNA using an RT reaction, followed by PCR amplification. Briefly, 5µl of the extracted RNA was assay in a 25µl reaction mixture. The thermal profile consisted of a reverse transcription step at 50°C for 30 min and the denaturation of RT enzyme at 95°C for 10 min, followed by the PCR reaction performed for three cycles at 94°C for 1 min, 60°C for 40 s, 72°C for 1 min, 35 cycles at 94°C for 30 s, 55°C for 30 s, and 72°C for 40 s and a final 72°C for 3 min. The PCR products were purified using the QIAquick PCR Purification kit (Qiagen, Germany) following the manufacturer’s protocol.

### Sequencing and Phylogenetic Analysis

Purified PCR products were directly sequenced in both forward and reverse directions using the same primers as for PCR using the ABI Prism® BigDye™ Terminator Cycle Sequencing Ready Reaction Kit (Applied Biosystems, USA) according to the manufacturer’s protocol. The nucleotide sequences were determined by ABI PRISM® 3700 Genetic Analyzer (Applied Biosystems). Sequences assemblies were completed using the SeqMan II software (DNASTAR, Inc., Madison, WI, USA) [16]. The identity of nucleotide and amino acid sequences was determined by use of the BLAST engine (http://www.ncbi.nlm.nih.gov/Entrez/). Nucleotide sequences were aligned with the sequence alignment software Clustal X v.1.83 [17]. The phylogenetic tree was constructed by the neighbour-joining methods with a Kimura 2 parameter model using MEGA 4.0 software (Molecular Evolutionary Genetics Analysis, Version 4.0) [18]. Bootstrap analyses with 1,000 replicates were used to determine the robustness of the tree. DENV-1 (D1/USA/Hawaii/1945) strains (GenBank accession number: AF425619) was used as out-group to root the trees.

## Results

### Index Cases Confirmation and Epidemics/Outbreaks Description

The first index patient, a businessman from Wenzhou, east China’s Zhejiang province, stayed in one hotel of Yuexiu district after returning from a business trip to Hu Chi Minh City, Vietnam, on February 28, 2009. On March 5, he suffered from a sudden fever (39°C), followed by cephalgia, malaise, arthralgia and exanthema on the legs and forearms. He fully recovered 6 days after onset without severe complications. Before leaving Canton, he visited Pazhou International Exhibition Center in Haizhu district twice on March 11 and March 12, respectively. On the basis of his symptoms and dengue prevalence in Vietnam, DF was confirmed and virus isolate 09/GZ/1081 (GenBank accession no. Hm466962) was found in his serum. On March 18, a worker at a construction site of the Pazhou International Exhibition Center, developed typical DF symptoms, including fever, headache, chills, rash, muscle and joint pain, and anorexia. Laboratory tests conducted on an early serum sample indicated negative serology for IgM and IgG antibodies but virus 09/GZ/1483 (GenBank accession no.466963) was isolated. He had no recent history of international travel. Consequently, the patient was considered of a confirmed autochthonous case of dengue virus infection.

The second index case was an India businessman who arrived at Canton on July 21, 2009. On July 22, he had a fever (38.5°C), showed fatigue and muscle and joint pain, and was admitted to a clinic in Dengfeng Street of Yuexiu district. Acute-phase blood samples (fever day 4) were taken and transported to the Canton CDC laboratory for serologic diagnosis and etiologic agent identification. After symptomatic treatment, he was discharged from the clinic on July 30 and rested in Dengfeng Hotel. On the basis of clinical and epidemiologic data, serum IgM- and IgG- antibody to dengue virus, and virus isolated 09/GZ/10616 (GenBank accession no.466964), his diagnosis was confirmed as DF. On July 31, he had a goods transaction in a market of Kuangquan Street in Yuexiu district. On August 6, 2009, three adult family members, living approximately 100 m from the goods transaction market which the second index case had been visited earlier, were admitted to Canton No.8 People’s Hospital as suspected DF cases. The 30-year-old son firstly had a sudden fever on August 4 with headache, then his father (56-year-old) and mother (50-year-old) fell ill subsequently in the following two days. The acute phase sera from all the three family members were positive for dengue IgM antibody, but negative for IgG antibody. Two viruses 09/GZ/11144 (GenBank accession no.Hm466966) and 09/GZ/11194 (GenBank accession no.Hm466967) were isolated. All cases recovered in a week post admission.

On April 25, 2010, a Tanzanian businessman had a sudden fever (38.2°C) two days after his arrival on Canton and was admitted to a clinic in Dengfeng Street on the next day. Interestingly, IgM and IgG were negative on disease day 2, and he was, therefore, misdiagnosed as having a viral infection of the upper respiratory tract. After anti-inflammatory treatment, he left the clinic on the same day and stayed at the Dengfeng Hotel until May 20. However, the acute-phase blood sample was stored at -80°C for the repeat screening for at least 30 days to meet the quality control requirements. On the basis of clinical and epidemiologic data and particularly virus isolation of 10/GZ/4898 (GenBank accession no.JN009093) from the stored specimen, his diagnosis was then changed to be DF on May 15. This case was confirmed to be the index of the dengue outbreak in Canton in 2010. This imported case were reported too late to implement vector control measures which routinely follow imported viraemic dengue cases. From May 12 to November 9, 2010, a total of 78 dengue cases were identified. None had traveled in the three weeks prior to disease onset to a dengue-endemic country. At the start, only sporadic cases surfaced around the area where the third index case had been living. Since August 5, cases were subsequently identified in other Canton districts, showing the spread of the virus. The cases concentrated emerging between September 16 and November 9, which accounted for 86.7% (52/60) of all dengue cases.

**Figure 1:**
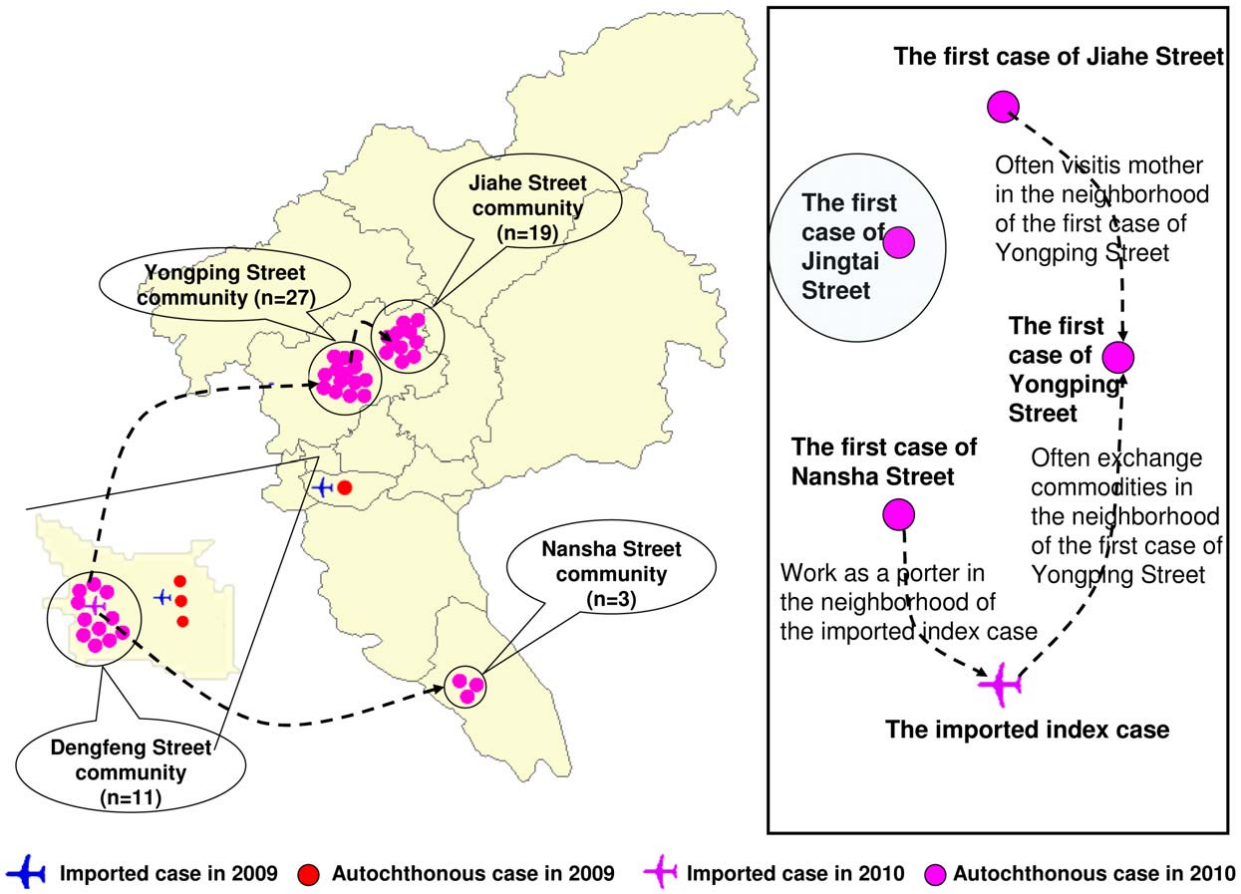
Geographical distribution and epidemiological links between the imported index case and corresponding autochthonous cases in the DENV-3 epidemics/outbreaks in Canton, during 2009–2010.

However, all these cases were confined to the Dengfeng Street community (n = 11), Nansha Street community (n = 3), Jiahe Street community (n = 19), Yongping Street community (n = 27), and Jingtai Street community (n = 18). With the exception of Jingtai Street community, epidemiological or geographical links were identified between all the first documented cases from each dengue affected community and the third index case (Figure 1), suggesting they were all related to a single chain of transmission. Further epidemiological and phylogenetic analyses showed that the virus responsible for the outbreak restrained to Jingtai Street community was DENV-4 genotype II and initially traced to the imported index case, a Canton resident who traveled back from Thailand [13]. Thus, only 60 cases epidemiologically linked to the third index case were included in present study.

### Clinical/Laboratory Diagnosis and Manifestations of Autochthonous Cases

**Figure 2:**
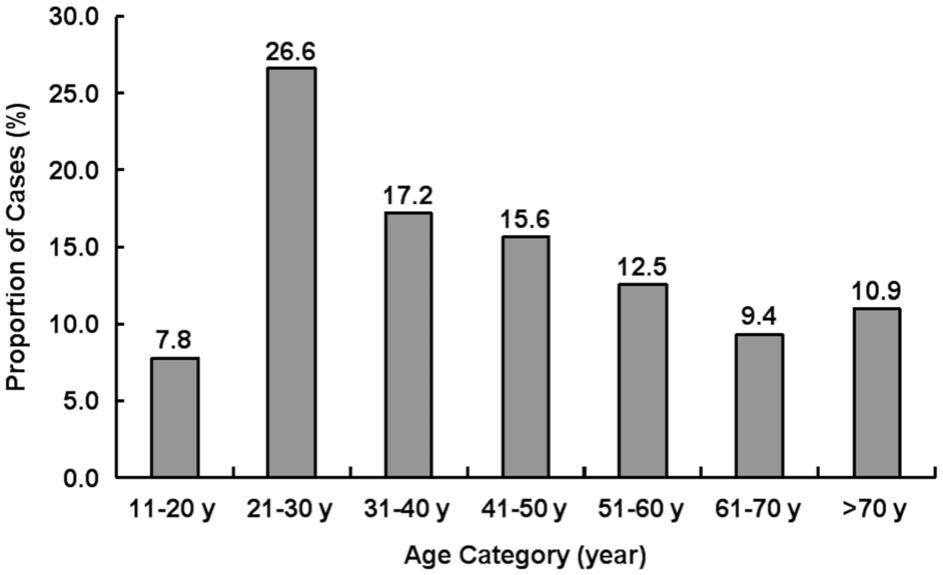
Age distribution of the 64 autochthonous dengue cases from the DENV-3 epidemics/outbreaks in Canton, during 2009–2010.

A total of 64 autochthonous cases were identified who met case definitions for laboratory-confirmed (54.7%) or clinically compatible (45.3%). Of these 64 cases, 61 cases were identified from passive reports when seeking medical services and 3 cases from active searches. No cases of DHF/DSS were found. The infected individuals consisted of 36 males and 28 females with sex ratio of 1∶1.29 and an average age of 40.36 years (range: 16–91 years). Cases in those aged 21 to 50 accounted for 59.4% of all cases (Figure 2). The clinical history revealed that all the patients had suffered from fever ranging from 38°C to 40°C. A summary of the clinical symptoms and signs is described in Table 1. Most of the common symptoms include malaise, headache, skin petechiae, rash, and myalgia. There was also a history of backache, conjunctival congestion, arthralgia, orbital pain, ficial flushing, cough, itch of skin, diarrhea, vomiting, and relative infrequent pulse. Clinical blood tests indicated leukocytopenia in 58 cases (90.6%) and thrombocytopenia in 43 cases (67.2%). The median duration of illness was 6 days (range: 2–14 days).

**Table 1:** Symptoms observed in the 64 autochthonous dengue cases in Canton, 2009–2010.

Symptoms	Number of cases	Proportion(%)
Fever	64	100.00
Leukocytopenia	58	90.63
Malaise	57	89.06
Headache	49	76.56
Skin petechiae	47	73.44
Rash	46	71.88
Thrombocytopenia	43	67.19
Myalgia	31	48.44
Backache	23	35.94
Conjunctival congestion	15	23.44
Arthralgia	11	17.19
Lymphadenectasis	7	10.94
Orbital pain	8	12.50
Facial flushing	9	14.06
Cough	6	9.38
Itch of skin	6	9.38
Diarrhea	5	7.81
Vomiting	4	6.25
Relative infrequent pulse	1	1.56

A total of 48 blood specimens were submitted with a median time interval from onset to sampling of six days (range 1–12 days). Thirty-one cases (64.6%) had IgM antibodies to DENV and 9 cases (18.8%) had IgG to DENV. Of 14 samples within six days after the onset of disease, ten (71.4%) (including the imported index cases) tested positive for dengue viral RNA by RT-PCR. Seven days after the onset of fever, viral RNA could not be detected. All cases had single DENV serotype infection and were typed as DENV-3. Of four 2010 endemic strains, one was isolated in Jiahe Street community (10/GZ/9721), two in Yongping Street community (10/GZ/9119 and 10/GZ/9534), and one in Nansha Street (10/GZ/5536). We have determined the complete *E* gene nucleotide sequences of the ten DENV-3 strains. All nucleotide sequences determined in this study have been deposited in GeneBank database with accession numbers listed in Table 2.

**Table 2:** Dengue virus type 3 strains newly isolated in Canton during 2009–2010.

Strain	Case	Date of Isolation	GenBank Accession No.	District	Geographical Origin
09/GZ/1081	Imported	2/28/2009	Hm466962	Yuexiu	Vietnam
09/GZ/1483	Endemic	3/18/2009	Hm466963	Haizhu	Canton
09/GZ/10616	Imported	7/25/2009	Hm466964	Yuexiu	India
09/GZ/11144	Endemic	8/6/2009	Hm466966	Yuexiu	Canton
09/GZ/11194	Endemic	8/6/2009	Hm466967	Yuexiu	Canton
10/GZ/4898	Imported	4/25/2010	JN009093	Yuexiu	Tanzania
10/GZ/5536	Endemic	5/12/2010	JN009094	Nansha	Canton
10/GZ/9119	Endemic	8/5/2010	JN009095	Baiyun	Canton
10/GZ/9534	Endemic	8/12/2010	JN009096	Baiyun	Canton
10/GZ/9721	Endemic	8/22/2010	JN009097	Baiyun	Canton

### Comparison of Nucleotide and Deduced Amino Acid Sequences

Envelope gene sequences of 7 isolates from the autochthonous cases and 3 isolates from the imported index cases were 1479nt in length, with nucleic acid homology ranged from 93.4% to as high as 100%. The percentages of nucleotide and amino acid identities among the strains isolated from autochthonous cases and their corresponding index cases are shown in Table 3. Pairwise comparisons of 5 DENV-3 isolates (imported strain-10/GZ/4898; endemic strains-10/GZ/5536, 10/GZ/9119, 10/GZ/9534 and 10/GZ/9721) from the 2010 epidemic area showed the highest similarity, with nearly 100% sequence identity in both nucleotide and amino acid sequences. The imported strain of 09/GZ/1081 showed 99.8% nucleotidic identity and 99.7% amino acid sequence homology relative to the corresponding endemic strain (09/GZ/1483). Two other endemic strains (09/GZ/11144 and 09/GZ/11194) shared 99.7% or more homology in both nucleotide and amino acid sequences with 09/GZ/10616 that was isolated from the second index case imported from India. However, sequence comparison of endemic strains resulting from different imported index cases showed higher sequence diversity (93.3%–98.5% nucleotide and 96.7%–96.9% amino acid sequence identity).

**Table 3:** Percentage identity within the complete E genomic sequences of 10 different DENV-3 strains obtained from Canton, during 2009–2010.

Pairwise amino acid identity (%)											
No.	Strain	1	2	3	4	5	6	7	8	9	10
1	10/GZ/4898	–	100	100	100	98.7	96.9	96.7	99.5	99.3	99.3
2	10/GZ/5536	100	–	100	100	98.7	96.9	96.7	99.5	99.3	99.3
3	10/GZ/9534	100	100	–	100	98.7	96.9	96.7	99.5	99.3	99.3
4	10/GZ/9721	100	100	100	–	98.7	96.9	96.7	99.5	99.3	99.3
5	10/GZ/9119	99.5	99.5	99.5	99.5	–	95.7	95.7	98.3	98.1	98.1
6	09/GZ/1081	93.4	93.4	93.4	93.4	93.0	–	99.7	97.3	97.1	97.1
7	09/GZ/1483	93.3	93.3	93.3	93.3	93.0	99.8	–	97.1	96.9	96.9
8	09/GZ/10616	98.7	98.7	98.7	98.7	98.3	938	93.7	–	99.7	99.7
9	09/GZ/11144	98.5	98.5	98.5	98.5	98.1	93.7	93.5	99.7	–	100
10	09/GZ/11194	98.5	98.5	98.5	98.5	98.1	93.7	93.6	99.7	99.9	–
**Pairwise nucleotide identity (%)**											

### Phylogenetic Analysis

A total of 90 DENV-3 strains available on GenBank and corresponding to strains belonging to all major branches of previously published DENV-3 phylogenies were included in this analysis and retrieved to maximize the representation of genotypes, regions and years of isolation. However, isolates that showed more than 99.5% nucleotide sequence homology but were adequately represented were removed from the analysis. Hence, the final tree only included representative local samples for each genotype.

**Figure 3:**
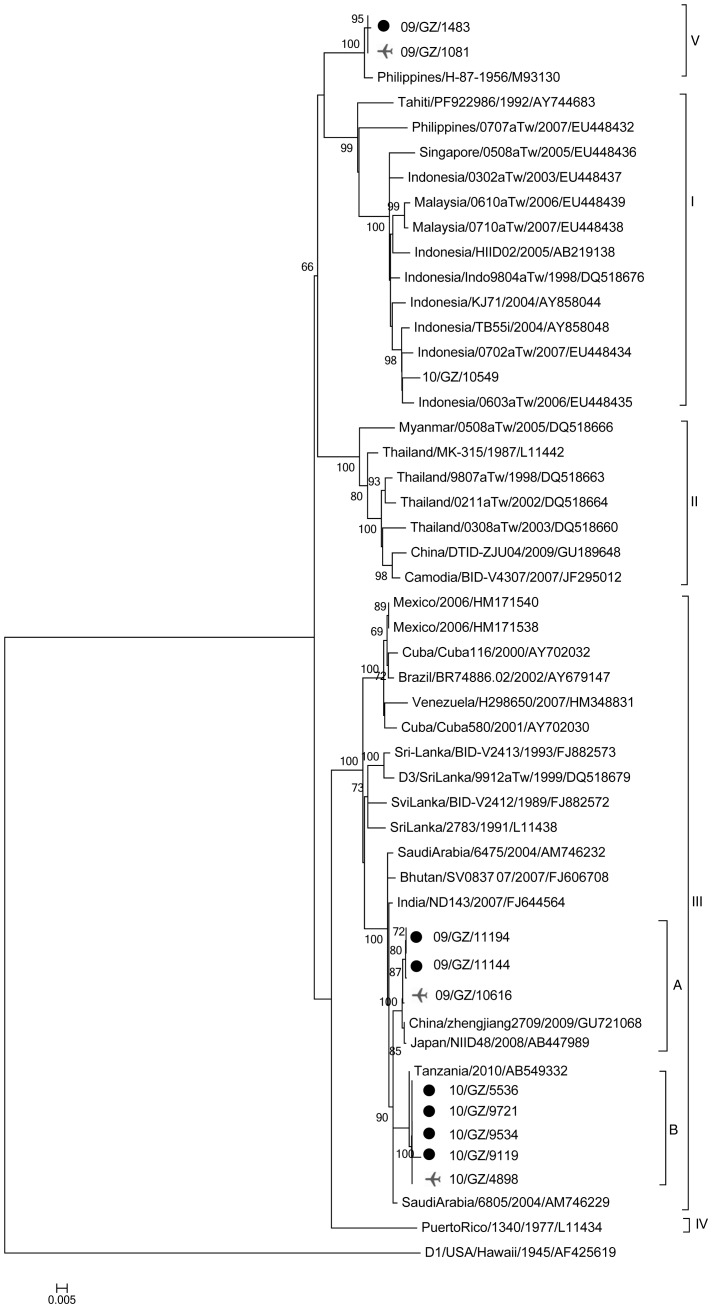
The evolutionary relationships of the envelope gene sequences of DENV-3. The phylogenetic tree was constructed by the neighbour-joining method with a Kimura 2 parameter model using MEGA 4.0 software. Bootstrap values were set for 1000 repetitions and were placed over each main node of the tree. The tree was rooted by strain of DENV-1 (AF425619).The black dots denoted endemic DENV-3 strains from Canton in 2009 and 2010. Airplane symbols denoted imported index DENV-3 strains from Canton in 2009 and 2010.

Phylogenetically, as demonstrated in Figure 3, five genetic subtypes of DENV-3, each representing a distinct genotype, were distinguished. Genotype I comprises viruses from Indonesia, Malaysia, Singapore, Philippines, and the South Pacific; Genotype II includes viruses from Thailand, Myanmar, Camodia, and China; Genotype III shows a wide geographical distribution including strains from Southeast Asia, Central & South America, Africa, and the Middle East; the most divergent group containing virus from Puerto Rico (genotype IV); and a final, represented by the oldest strain isolated in the Philippines in 1956 (genotype V).

The DENV-3 strains newly isolated in Canton during 2009–2010 epidemics/outbreaks were grouped into 2 genotypes (III and V). The first endemic strain (09/GZ/1483) and the corresponding index strain (09/GZ/1081) imported from Vietnam belonged to the genotype V. Among the Canton genotype III strains, two clades can be identified with three 2009 isolates (imported strain: 09/GZ/10616; endemic strains: 09/GZ/11144 and 09/GZ/11194) clustered in clade-A and all five 2010 isolates forming clade-B.

## Discussion

The re-emergence of DENV-3 in Canton in 2009 and again in 2010 occurred after a decade of active DENV-1 circulation and 30 years after its first introduction in 1980. Understanding the factors that contributed to the re-emergence of DENV-3 after such a prolonged absence will help public health authorities develop practicable prevention and control strategies. Like previous epidemics/outbreaks, the 2009 epidemics and 2010 outbreaks also mainly struck following the monsoon season, when the climatic factors (temperature and humidity) remained conducive for *Aedes* breeding [7]. During these epidemics/outbreaks it has been observed that majority of the patients belonged to age group of 21–50 years, which is contrary to the popular belief that dengue is a paediatric disease [19], [20], [21].

Serology testing for dengue virus-specific antibodies, types IgG and IgM, can be useful in confirming primary or secondary diagnosis [22]. Notably, on the basis of the results of a recent laboratory study, IgM is produced approximately 5 days after infection in both primary and secondary infections, while IgG is produced about 2 to 4 weeks after onset of primary infection and can not be detected in samples collected on disease day 10 or earlier, but almost immediately after onset of a secondary infection [23]. In this study, approximately 19% of patients presenting with the IgG antibodies on disease days 5 to 10 revealed that they might be suffering from secondary dengue infection. However, in non-endemic areas, such as Canton, the risk of dengue is largely driven by travel to endemic areas, as is indicated in present study. Particularly, all of the IgG-positive individuals belonged to the age group of 23–54 years (median 41). Thus, we deduced that the secondary dengue infection may be due largely to travel.

To trace the sources of infection, epidemiologic studies and phylogenetic analyses were preformed in the cohort of infected persons. Epidemiologic data strongly suggested that the first autochthonous case was directly linked to the first index case who returned from Vietnam. Phylogenetic analysis showed that the isolate (09/GZ/1483) of the first autochthonous case and the DENV-3 strain (09/GZ/1081) from the first index case shared 99.8% nucleotidic identity and were grouped together in the same clade which belonged to DENV-3 genotype V. Genotype V was seldom associated with dengue epidemics and is only represented by a few early sequences from the Asia, with the exception of recent reports from Brazil and Colombia [24], [25]. Despite an active search of symptomatic cases among their close contacts and in the neighbourhood, no other cases were identified. On the one hand, a lack of secondary transmission may be evidence that January to May has only sporadic cases occurrence due to drier and cooler weather [5]. On the other hand, it may also associated with the lower epidemic potential with regard to severe dengue epidemics of genotype V [24], [25], [26], [27].

Although the phenomenon of family cluster of vector-borne diseases have been intensively described [28], [29], information regarding dengue fever family cluster is limited. During the other 2009 dengue epidemic in Canton, a family clustering of DENV-3 infections was described. Three family members were diagnosed as dengue fever, all recalled mosquito biting before illness, and none of them went abroad or on trips recently. This classification prompted an immediate reaction of public health authorities to reduce the risk of further spread of the virus. No further cases were reported nearby thereafter. According to results described previously [30], two strains (09/GZ/11144 and 09/GZ/11194) isolated from the three family members were local circulating strains. But in present study, both strains shared 99.7% nucleotidic identity with the 09/GZ/10616 strain isolated from the second index case imported from India and epidemiological links were identified between them. Phylogenetically, 09/GZ/10616, 09/GZ/11144 and 09/GZ/11194 are located in the same clade formed by strains isolated in 2008 from a Japanese traveler to Côte d’Ivoire (Japan/NIID48/2008/AB447989). Therefore, we concluded that this dengue epidemic was also caused by an imported case.

On comparison of the sequence, we also found that the outbreaks in Canton and Zhejiang Province (more than 1400 km apart) in 2009 were caused by the same type of DENV-3. It shows that this clade of DENV-3 subtype III can be easily transmitted and can adapt efficiently to new areas. Thus, other regions where the climate is similar to that of Canton and Zhejiang Province (subtropical monsoon) should be aware of the risk for expansion of these strains [12].

We also determined the genetic relationship and origin of the DENV responsible for the 2010 dengue outbreak in Canton. Based on epidemiological and phylogenetical analyses, this current outbreak was confirmed as DENV-3 genotype III and initially traced to the imported index case from Tanzania. All four 2010 Canton endemic strains and their corresponding imported strains were identical and formed a close branch along with AB549332 from Tanzania, which corresponded with the index case’s travel history. The DENV-3 genotype III involved in 2010 outbreak spread rapidly, but was not classified as a re-emergence of the 2009 strains. However, both (Clade-A and Clade-B) clades appeared to share a common ancestral lineage with a 2004 isolate (SaudiArabia/6805). Clearly, these two clades evolved separately from a common ancestor originating from the Saudi Arabia.

Recently, it is believed that the dengue outbreaks in Canton were increasingly becoming endemic infections of DENV circulating locally [15], [30], [31]. However, all present findings implicated that repeated introductions of different DENV-3 strains were one of the important causes of the 2009 epidemics and 2010 outbreak in Canton. Our epidemiological and biogeographical analyses identified at least three plausible routes of entrance of DENV-3 into Canton. The first route was from Vietnam, the second one from India, and the third one from Tanzania. However, DENV-3 genotypes, particularly the genotype III, have demonstrated their higher potential to spread, adapt and dominate in geographically diverse areas of the world [32]. This subtype has also been implicated in the emergence of severe epidemics involving more DHF cases [33], [34]. Once the transmission cycle established locally, the genotype III may result in dengue epidemics, even a trans-national dengue pandemic [27], [35], [36]. The change in the predominant serotype from DENV-1, which had been circulating for at least 8 years uninterruptedly, to DENV-3 is also a matter deserving great concern due to the immunologically naive to the new circulating serotype.

Several reasons for the 2010 outbreak of dengue in Canton should be considered. First, it was triggered by an imported case which was initially misdiagnosed. Early diagnosis of dengue is challenging because the initial symptoms are often non-specific and viraemia may be below detectable levels [37]. The misdiagnosis at the early stage might have contributed to a higher chance of the subsequent outbreak. Second, Cantonese life habits of culturing water plants and keeping water for watering flowers, and environment were suitable for the breeding of *Aedes albopictus*. Finally, lack of self-awareness of protection against dengue greatly contributed to its subsequent transmission.

Consequently, the link between imported cases and autochthonous cases has been established through both epidemiological and phylogenetical analyses. To identify imported dengue cases and reduce the local spread of newly introduced dengue viruses, an integrated dengue control program including various surveillance systems, a network of rapid diagnostic laboratories, and mechanisms of rapid response to implement control measures has been established by the government of Canton. As part of the integrated dengue control program, fever screening at international airports (identifying fever cases by infrared thermal scanner) has been reported to be an effective means of identifying imported dengue cases [38], [39]. However, in Canton, for febrile passengers suspected of having dengue virus infection, they are neither detained at the airport nor required to complete the “Dengue Survey Form”, and only provided with a mosquito net. Most people do not use it at all due to inconvenience. Thus, its cost-effectiveness in preventing any dengue epidemics in Canton will need to be re-evaluated.

### Conclusions

In summary, we concluded that: (i) the re-emergence of DENV-3 in Canton during 2009–2010 were caused by imported cases; (ii) DENV-3 from 2009–2010 were not from continuous spread of the same epidemic strain or re-emergence of the 2009 strains in the 2010 period; (iii) more than one plausible route of entrance of DENV-3 into Guangzhou exists. This study has re-affirmed the importance of a surveillance system for dengue control and aroused the awareness of the role of imported cases in the onset of epidemics/outbreaks.
